# The association of permanent versus absorbable fixation on developing chronic post-herniorrhaphy groin pain in patients undergoing laparoscopic inguinal hernia repair

**DOI:** 10.1007/s00464-024-10866-z

**Published:** 2024-05-06

**Authors:** Kimberly P. Woo, Ryan C. Ellis, Sara M. Maskal, Daphne Remulla, Priya Shukla, Alexandra J. Rosen, Isabella Wetzka, Wilhemina Osei-Koomson, Sharon Phillips, Benjamin T. Miller, Lucas R. Beffa, Clayton C. Petro, David M. Krpata, Ajita S. Prabhu, Emanuele Lo Menzo, Michael J. Rosen

**Affiliations:** 1 Department of General Surgery, Digestive Disease Institute, Cleveland Clinic, 9500 Euclid Ave, Cleveland, OH 44195 USA; 2 Department of Biostatistics, Vanderbilt University Medical Center, Nashville, TN USA; 3 Department of General Surgery, Bariatric and Metabolic Institute, Cleveland Clinic Florida, Weston Hospital, Weston, FL USA

**Keywords:** Inguinal hernia, Tack, Fixation, Groin pain

## Abstract

**Introduction:**

Fixation of mesh during minimally invasive inguinal hernia repair is thought to contribute to chronic post-herniorrhaphy groin pain (CGP). In contrast to permanent tacks, absorbable tacks are hypothesized to minimize the likelihood of CGP. This study aimed to compare the rates of CGP after laparoscopic inguinal hernia repair between absorbable versus permanent fixation at maximum follow-up.

**Methods:**

This is a post hoc analysis of a randomized controlled trial in patients undergoing laparoscopic inguinal hernia repair (NCT03835351). All patients were contacted at maximum follow-up after surgery to administer EuraHS quality of life (QoL) surveys. The pain and restriction of activity subdomains of the survey were utilized. The primary outcome was rate of CGP, as defined by a EuraHS QoL pain domain score ≥ 4 measured at ≥ 1 year postoperatively. The secondary outcomes were pain and restriction of activity domain scores and hernia recurrence at maximum follow-up.

**Results:**

A total of 338 patients were contacted at a mean follow-up of 28 ± 11 months. 181 patients received permanent tacks and 157 patients received absorbable tacks during their repair. At maximum follow-up, the rates of CGP (27 [15%] vs 28 [18%], *P* = 0.47), average pain scores (1.78 ± 4.38 vs 2.32 ± 5.40, *P* = 0.22), restriction of activity scores (1.39 ± 4.32 vs 2.48 ± 7.45, *P* = 0.18), and the number of patients who reported an inguinal bulge (18 [9.9%] vs 15 [9.5%], *P* = 0.9) were similar between patients with permanent versus absorbable tacks. On multivariable analysis, there was no significant difference in the odds of CGP between the two groups (OR 1.23, 95% CI [0.60, 2.50]).

**Conclusion:**

Mesh fixation with permanent tacks does not appear to increase the risk of CGP after laparoscopic inguinal hernia repair when compared to fixation with absorbable tacks. Prospective trials are needed to further evaluate this relationship.

In the USA, an estimated 800,000 inguinal hernia operations are done annually [1], with up to 1 in 4 men and 1 in 30 women undergoing a repair in their lifetime [2]. With the advent of minimally invasive surgery, the trend has shifted toward laparoscopic approaches [3, 4], such as the totally extraperitoneal (TEP) or trans-abdominal preperitoneal (TAPP) repair techniques, both of which commonly include placement and fixation of mesh. While postoperative complications are rare, post-herniorrhaphy groin pain is a relatively common adverse outcome.

However, the etiology of this pain is not well understood and is likely multifactorial in nature. Age, gender, BMI, preoperative pain level, hernia size, presence of postoperative complications and history of prior inguinal hernia repair have all previously been identified as potential predictors for postoperative pain [5–10]. Additionally, there exists controversy surrounding the impact of fixation on post-herniorrhaphy groin pain. Permanent titanium tacks have historically been used, but a number of reported complications [11, 12] and their possible association with severe postoperative pain [13, 14] has generated concern regarding the type of tacks that should be used for fixation. Though few studies have definitively identified a significant effect of permanent fixation on pain [15], the theoretical concern has led to the development of more costly alternatives, such as absorbable tacks.

The effect of permanent versus absorbable fixation on chronic post-herniorrhaphy groin pain after inguinal hernia repair has been assessed in only a handful of studies with small patient cohorts [16, 17]. Comparatively, it has been evaluated in more studies in incisional or ventral hernias. The data there has not shown an increased risk of chronic post-herniorrhaphy groin pain with permanent tacks [18–22] and therefore, no conclusive data exist to justify the use of the more expensive absorbable tacks for fixation. Still, randomized controlled trials were relatively small and lacked long-term follow-up of pain. We aim in this study to compare the rates of chronic post-herniorrhaphy groin pain after laparoscopic inguinal hernia repair between permanent versus absorbable tack fixation at maximum follow-up of a large cohort of patients.

## Methods

### Patient population and data source

This study is a long-term follow-up and post hoc analysis of a multi-center, single-blinded, parallel group RCT (NCT03835351). The original trial design and outcomes were previously reported [23]. In brief, patients 18 years or older, undergoing laparoscopic, elective inguinal hernia repair were assessed for the effect of intraoperative urinary catheters on postoperative urinary retention. The trial was conducted at six academic and community hospitals with 11 participating surgeons from March 2019 to March 2021. Institutional Review Board (IRB) approval was granted at participating sites before enrollment and all study participants provided written informed consent. Patients unable to provide written informed consent or unable to tolerate general anesthesia were excluded.

All operations were performed by general surgeons with advanced training in minimally invasive inguinal hernia repair. Surgical technique and method of mesh fixation were left to the discretion of the operating surgeon. Permanent fixation was achieved with ProTack™ (Covidien, Minneapolis, MN) titanium tacks, while absorbable fixation was achieved with AbsorbaTack™ (Covidien, Minneapolis, MN).

IRB approval was obtained for the current post hoc analysis. All patients who completed the previous RCT were eligible for inclusion in this analysis. These patients were contacted at maximum follow-up after surgery to obtain EuraHS Quality of Life (QoL) and Hernia Recurrence Inventory (HRI) assessments. EuraHS is a hernia-specific QoL instrument, validated for the perioperative assessment of inguinal repair outcomes [24]. Patients score their symptoms from a range of 0 to 10 with 10 indicating the most severe effect and interpreted as worse QoL. HRI is a patient-reported outcome (PRO) questionnaire that consists of three questions: (1) Do you think your hernia has come back? (2) Do you feel or see a bulge? (3) Do you have pain or physical symptoms at the site. The question “Do you feel or see a bulge?” has previously been demonstrated to be highly sensitive and specific for the diagnosis of an inguinal hernia recurrence [25] and was used in this study to measure the rates of patient-reported hernia recurrence.

### Outcome of interest

Two subdomains of the EuraHS-QoL survey were included in this analysis: pain and restriction of activities. The pain domain ranges from 0 to 30 and restriction of activities from 0 to 40. The previously validated minimum clinically important differences (MCID) for each domain are 3 for the pain domain and 5 for the restriction of activity domain [26]. The primary outcome was rate of chronic post-herniorrhaphy groin pain, as defined by a EuraHS-QoL pain domain score ≥ 4 measured at maximum follow-up (at least 1 year) after surgery. A cutoff score that is indicative of persistent pain has yet to be validated for this instrument. Therefore, a score of ≥ 4 was identified based on previously employed quantitative definitions in the chronic pain literature [27–30]. Additional outcomes from the EuraHS-QoL survey were average pain and restriction of activity domain scores and HRI patient-reported hernia recurrence at maximum follow-up. Multivariable analysis was performed adjusting for possible risk factors of chronic post-herniorrhaphy groin pain, including age, BMI, hernia size, and number of tacks. Number of tacks was analyzed as a categorical variable, ≤ 4 tacks vs > 4 tacks. Given the standard for mesh fixation is four tacks, this cutoff was chosen to ensure the variable reflected increasing tack type from the norm.

### Statistical analysis

Continuous variables were compared using Wilcoxon rank sum test and categorical variables were compared using Pearson Chi-square or Fisher’s exact test where appropriate. Multivariate analysis was conducted using logistic regression. A *P* value < 0.05 was considered statistically significant. All statistical analyses were conducted using R software (version 4.2.2, Vienna, Austria).

## Results

Of the 496 patients in the original RCT who were randomized, 486 patients (98%) remained at the conclusion of the trial and were eligible for inclusion in the current study. All eligible patients were contacted, and follow-up was successfully obtained for a total of 345 patients at a mean follow-up of 28 ± 11 months. Operative details, including fixation type, were not available for seven patients. Therefore, they were excluded, and 338 (70%) patients were included in the statistical analysis.

### Patient and operative characteristics

Mesh fixation for inguinal hernia repair was achieved with permanent tacks in 181 patients (53.6%) and absorbable tacks in 157 patients (46.4%). The baseline characteristics and comorbidities between these two groups were similar (Table 1). The median (IQR) age of patients who received permanent fixation was 61 (54–67) years and 62 (50–70) years for absorbable fixation. The baseline median (IQR)-reported pain scores were also similar (*P* = 0.54); 6 (2–12) for patients with permanent fixation and 8 (3–11) for patients with absorbable fixation. However, baseline EuraHS-QoL surveys were not collected from all patients in the original RCT and were only available for 229/338 (67%) patients in this analysis.

**Table 1:** Patient demographics and comorbidities

	*n*	Permanent fixation *n* = 181	Absorbable fixation *n* = 157	*P* value
**Age (years), median (IQR)**	338	61 (54, 67)	62 (50, 70)	0.69
**Sex**	338			0.70
Female		6.1% (11)	5.1% (8)	
Male		93.9% (170)	94.9% (149)	
**BMI**	335			0.09
≤ 29.9		79.3% (142)	82.7% (129)	
30–34.9		14.5% (26)	16.0% (25)	
35–39.9		5.6% (10)	0.64% (1)	
≥ 40		0.6% (1)	0.64% (1)	
**ASA class**	335			0.37
1		12.3% (22)	12.8% (20)	
2		54.7% (98)	62.8% (98)	
3		32.4% (58)	23.7% (37)	
4		0.6% (1)	0.6% (1)	
**Diabetes**	335	4.5% (8)	5.8% (9)	0.59
**Hypertension**	338	38% (68)	29% (46)	0.11
**COPD**	335	1.7% (3)	0.0% (0)	0.10
**Current smoker**	335	6.2% (11)	5.8% (9)	0.88
**Baseline EuraHS QOL scores**				
Pain domain, median (IQR)	229	6 (2, 12)	8 (3, 11)	0.54
Restriction of activity domain, median (IQR)	227	9 (1.5, 18)	11 (2, 20)	0.22

The perioperative characteristics are listed in Table 2. More of the patients who received permanent fixation had previously undergone hernia repair with mesh than those who received absorbable fixation (12 [15.4%] vs 2 [3.3%], *P* = *0*.023). Patients with permanent fixation tended to have hernias that were > 3 cm or > 2 fingertips compared to those with absorbable fixation (53 [29.8%] vs 26 [16.8%], *P* = 0.01). For the operative approach, 103 patients (30.9%) underwent TAPP and 230 (69.1%) underwent TEP inguinal hernia repair. Absorbable tacks were used almost exclusively in the TEP approach (153 [98.7%]), whereas permanent tacks were used almost equally between TAPP and TEP repairs (101 [56.7%] and 77 (43.3%)]. More patients undergoing repair with permanent fixation received greater than four tacks for mesh fixation compared to those with absorbable fixation (110 [61.8%] vs 7 [4.5%], *P* < 0.001).

**Table 2:** Perioperative characteristics

	*n*	Permanent fixation *n* = 181	Absorbable fixation *n* = 156	*P* value
**Prior inguinal mesh**	139			0.02
No		84.6% (66)	96.7% (59)	
Yes		15.4% (12)	3.3% (2)	
**Hernia size**	333			0.01
≤ 3 cm or ≤ 2 fingertips		70.2% (125)	83.2% (129)	
> 3 cm or > 2 fingertips		29.8% (53)	16.8% (26)	
**Laterality**	333			0.92
Bilateral		38.2% (68)	38.7% (60)	
Unilateral		61.8% (110)	61.3% (95)	
**Surgical repair approach**	333			< 0.001
TAPP		56.7% (101)	1.3% (2)	
TEP		43.3% (77)	98.7% (153)	
**Number of tacks**	333			< 0.001
≤ 4		38.2% (68)	95.5% (147)	
> 4		61.8% (110)	4.5% (7)	
**Operation time**	335			< 0.001
< 1 h		28.5% (51)	46.8% (73)	
≥ 1 h		71.5% (128)	53.2% (83)	

### Patient-reported outcomes: EuraHS-QoL and HRI

Average follow-up for the full cohort was 26 months; 27 months for permanent fixation and 26 months for absorbable fixation (*P* = 0.41). Maximum follow-up was collected at 1 year for 62 patients (18.3%), at 2 years for 146 patients (43.2%), at 3 years for 122 patients (36.1%), and at 4 years for eight patients (2.4%). At maximum follow-up, the mean EuraHS-QoL pain domain scores were not statistically different between the patients who received permanent versus absorbable fixation (1.78 ± 4.38 vs 2.32 ± 5.40, *P* = 0.22). The restriction of activity domain scores were also similar (1.39 ± 4.32 vs 2.48 ± 7.45, *P* = 0.18). Tack type did not influence patient-reported hernia recurrence; the number of patients who reported an inguinal bulge was similar between permanent and absorbable fixation (18 [9.9%] vs 15 [9.5%], *P* = 0.9).

### Chronic post-herniorrhaphy groin pain and predictors

**Fig. 1:**
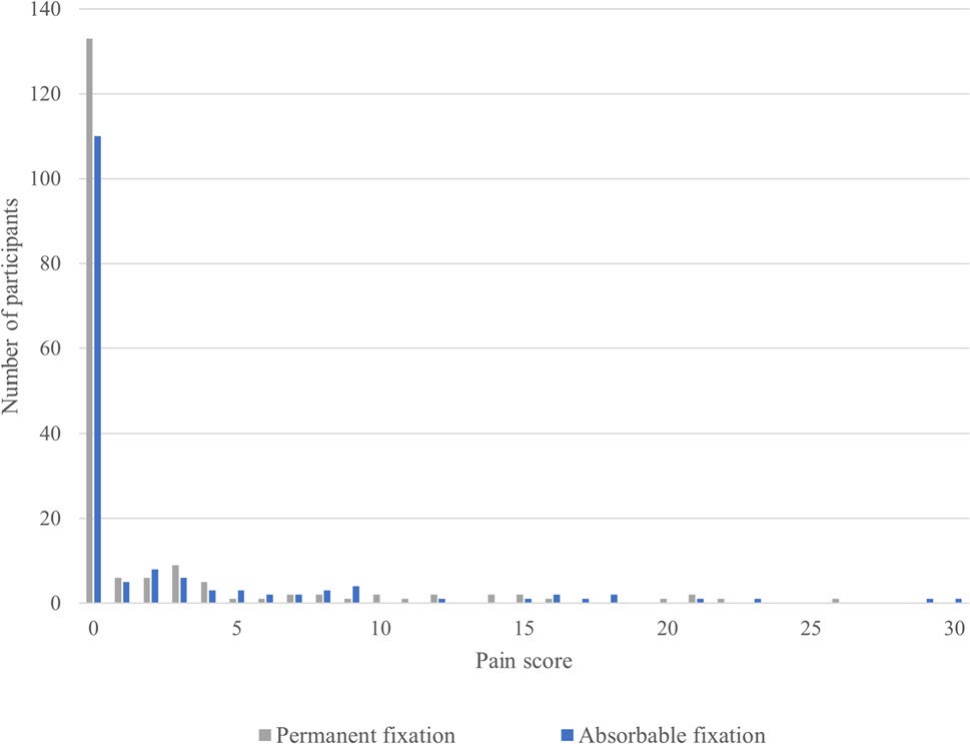
Pain scores at maximum follow-up

The overall rate of chronic post-herniorrhaphy groin pain (pain domain score ≥ 4) at maximum follow-up was 16.3% (Fig. 1). There was no statistically significant difference in the rates between permanent and absorbable fixation (27 [15%] vs 28 [18%], *P* = 0.47). On multivariable analysis, age, BMI, hernia size, and number of tacks used were not associated with chronic post-herniorrhaphy groin pain (Table 3). Though patients with absorbable fixation had 1.3 times odds of having chronic pain at maximum follow-up compared to patients with permanent fixation, this did not reach statistical significance (95% CI 0.60, 2.50).

**Table 3:** Predictors of chronic post-herniorrhaphy groin pain at maximum follow-up

	OR (95% CI)	Significance (*P*)
**BMI**	0.96 (0.71–1.30)	0.79
**Age**	0.94 (0.76–1.16)	0.59
**Tack type**		
Permanent	1.0 (Reference)	0.57
Absorbable	1.23 (0.60–2.50)	
**Hernia siz**e		
≤ 3 cm	1.0 (Reference)	0.49
> 3 cm	1.27 (0.64–2.50)	
**Number of tacks used**		
≤ 4 tacks	1.0 (Reference)	0.71
> 4 tacks	0.87 (0.40–1.88)	

## Discussion

Inguinal hernia repair is a very common and relatively safe procedure with minimal complications. However, some patients may experience persistent pain after surgery, known as chronic post-herniorrhaphy groin pain, with the type of tack used for mesh fixation thought to be a contributing factor. In this post hoc analysis of patients undergoing laparoscopic inguinal hernia repair, we did not identify any difference in the pain scores at maximum follow-up or in the rates of chronic post-herniorrhaphy groin pain between absorbable versus permanent tack fixation.

The rates of chronic post-herniorrhaphy groin pain vary widely in the literature, from 11 to 53% [6–9, 31–35]. This is generally considered to be due to the lack of a uniform definition or standardized tool for assessment. The International Association for the Study of Pain (IASP) defines chronic postsurgical pain as pain that “develops or increases in intensity after a surgical procedure” and “persists beyond the healing process, i.e., at least 3 months after the surgery” [36]. Most studies of chronic pain cite this definition, with collection of data occurring at time points of six months or greater [6, 7, 31–33]. Our rate of chronic post-herniorrhaphy groin pain at 1 year or greater falls within the range of published data, though on the lower end of the spectrum. This can potentially be attributed to how pain was defined and assessed for in our analysis. A closer review of the literature reveals that our rate of 16.3% is consistent with rates of approximately 11–12% that were calculated from pooled analyses [34, 35] and with other questionnaire-based studies that employed quantitative cutoffs for reported pain [32, 33]. This is in contrast to studies that found higher rates but defined chronic pain as simply the presence of pain to some extent [6–8, 31]. By establishing a cutoff, we aimed to identify groin pain that was clinically relevant and more likely to impact patient quality of life.

Given its relatively high incidence, many studies have aimed to identify the risk factors associated with the development of chronic post-herniorrhaphy groin pain. Younger age and recurrent hernias have consistently been found to be independent risk factors for chronic pain [5–9, 31, 34]. Despite our permanent fixation cohort having a significantly greater proportion of patients with prior inguinal mesh, the rates of patients reporting chronic groin pain did not differ compared to the absorbable fixation cohort. Other risk factors, such as smaller hernia defect size [37], increased BMI [10], or increased number of tacks used for fixation [38] have also been implicated in the development of chronic pain. None of these variables were found to be statistically significant in our multivariate analysis. Of note, it has also been demonstrated that high levels of preoperative pain serve as a risk factor for chronic pain [6, 7, 39]. There were, unfortunately, insufficient baseline pain scores available to reliably include this variable in our multivariate analysis.

While no studies on chronic post-herniorrhaphy groin pain have identified tack type as an independent risk factor, there have been only a few studies, limited by small sample sizes, directly comparing the impact of permanent versus absorbable tacks in inguinal hernia repairs. This was evaluated in 2018 by Hany et al*.* [16] in an RCT of 30 patients undergoing TAPP repair. At six months and at 1 year, there was no statistically significant difference in the pain scores between patients with permanent versus absorbable fixation. At a mean follow-up of 12 months, there were no recurrences in any of their patients. This study was followed in a case–control study of 20 patients undergoing TEP repair by Prakash et al*.* [17] in 2019. They too found no difference in pain scores at any of the follow-up time points ranging from six hours to six months postoperatively and no recurrences were identified with the mean follow-up of 24 months. Consistent with these two prior studies, we did not find any difference between the two fixation types in the patient-reported pain scores or in the rates of chronic pain as previously defined. No other prospective studies with larger patient cohorts have been published comparing the two types of tacks. To our knowledge, our post hoc analysis is one of the largest studies of any type evaluating the impact of tack type on chronic post-herniorrhaphy groin pain after inguinal hernia repair.

Despite the lack of prospectively collected data in inguinal hernias, there have been several RCTs comparing outcomes of absorbable versus permanent tack fixation in ventral or incisional hernia repairs. In one of the earliest RCTs comparing the two types of tacks for fixation, Colak et al*.* [18] found no difference in the six-month pain scores of 51 patients. At a median follow-up of 31 months, there was also no difference in rates of recurrence. Two subsequent RCTs similarly concluded that permanent tacks did not increase the risk of chronic post-herniorrhaphy groin pain or rates of recurrence [19, 20]. Interestingly, two retrospective analyses [21, 22] found that while the rates of chronic pain were the same, early postoperative pain was found to be significantly decreased in patients receiving absorbable tack fixation. The authors posited that the lack of deep penetration into bony structures of the absorbable tacks could explain this relative decrease in pain on postoperative days 1 and 10 [21]. Nevertheless, a difference in early postoperative pain was not supported in any of the randomized trials [18–20]. Even with the compelling evidence showing no association between tack type and chronic pain in ventral hernia repairs, the differences in anatomy between the abdominal wall and groin make this data alone insufficient to conclude the same in inguinal hernia repairs.

Importantly, as healthcare spending in the USA continues to rise, there has been a lack of demonstrable improvement in quality of care and outcomes [40]. With inguinal hernia repairs, the trend toward laparoscopic, and now robotic, approaches increases cost without value added [41]. The tendency to employ newer, more expensive technology and devices without evidence of superiority further adds to this burden. While costs can vary, at our institution, permanent fixation with ProTack™ is approximately $200 less than the cost of absorbable fixation with AbsorbaTack™. Our finding of similar outcomes between the two types of fixation, consistent with prior published literature, provides little justification to employ the more costly option.

While acknowledging the benefits of the large sample size in this analysis, there are some limitations that deserve mention. Because this was a post hoc analysis, there may have been a selection bias in the decision of which patients were enrolled in the original RCT. Importantly, though, the inclusion criteria of the trial were very broad [23], and the CONSORT diagram indicates that only a small proportion of patients (38/534, 7.1%) undergoing inguinal hernia repair during the enrollment period were excluded from the trial. Perhaps more significantly, follow-up was only successfully obtained in 70% of eligible patients. This leaves the possibility this missing data could have impacted the results of our study. We therefore performed a sensitivity analysis assuming a minimum pain score of 0 for all missing patients and a maximum pain score of 30 for all missing patients. Neither of these analyses yielded significant associations between pain at maximum follow-up and tack type. As previously stated, preoperative pain has been implicated as a significant predictor for the development of postoperative chronic pain. However, incomplete collection of baseline pain scores precluded the ability to reliably include this variable in our multivariate analysis. Our selected definition for chronic pain may not have been powered to detect smaller differences in rates of patients experiencing chronic pain between the two types of mesh fixation. While the HRI has been validated for the detection of recurrent inguinal hernias, it has not been specifically studied in the obese population. The ability to identify a recurrence may have been limited in this group of patients within our study population. And finally, the retrospective nature of our analysis precluded the ability to guarantee data collection at specific time points. Therefore, our outcomes were measured at maximum follow-up, thereby requiring uniform analysis of a potentially heterogeneous population.

In conclusion, the fixation of mesh with permanent tacks does not appear to increase the risk of chronic post-herniorrhaphy groin pain after laparoscopic inguinal hernia repair when compared to fixation with absorbable tacks. Appropriately powered prospective trials are needed to further evaluate this relationship.
